# Platelet Membrane-Camouflaged Biomimetic Drug Delivery Systems for Ischemia-Reperfusion Injury: Targeted Therapeutic Strategies

**DOI:** 10.33549/physiolres.935585

**Published:** 2025-08-01

**Authors:** Chun SHI, Tong LI, Bensi ZHANG

**Affiliations:** 1 College of Dental Medicine, Western University of Health Sciences, Pomona, CA, USA; 2 Department of Anatomy, Dali University, Dali, Yunnan, China

**Keywords:** Platelet Membranes, Drug delivery systems, Ischemia-reperfusion injury

## Abstract

This review summarizes the application of platelet membrane-coated biomimetic drug delivery systems in the treatment of various ischemia-reperfusion injuries (I/RI). Platelet membrane-coated nanoparticles, extracellular vesicles, microbubbles, microemulsions, as well as platelet membrane vesicles and their derivatives have shown significant promise for I/RI treatment. By leveraging the specific targeting, adhesive, and immune evasion properties of platelets, these systems enable the targeted delivery of therapeutic agents, such as antioxidants, anti-inflammatory agents, thrombolytics, and neuroprotective compounds, to ischemic tissues, while also offering diagnostic capabilities. However, challenges remain in bridging the gap between research and clinical application, such as scalability, maintaining bioactivity, and potential side effects. Future research should focus on improving scalability, stability, and safety. Furthermore, the context-specific selection or integration of delivery strategies is essential to meet the therapeutic demands of I/RI.

## Introduction

Ischemia-reperfusion injury (I/RI) is a pathological condition in which cellular dysfunction and cell death paradoxically worsen after blood flow is restored to ischemic tissues [1]. This process starts with ischemia-induced oxygen deprivation and metabolic disturbances, followed by reperfusion, which triggers oxidative stress, inflammation, complement activation, and leukocyte infiltration [1–2]. These events lead to endothelial injury, increased permeability, and subsequent organ dysfunction [1].

Given the complex nature of I/RI, effective treatment strategies are essential. Traditional therapies often face challenges such as poor bioavailability, rapid clearance, and difficulty in reaching ischemic tissues. Additionally, systemic administration can often cause off-target effects and toxicity. Biomaterial-based drug delivery systems, particularly those that integrate synthetic and natural materials like cell membranes, offer a biomimetic approach to address these limitations [3–4]. By enhancing drug stability and enabling precise targeting, these systems improve drug delivery and molecular imaging while minimizing systemic cytotoxicity [3–4]. The cell membrane coating strategy further enhances these benefits by providing surface modifications, extended circulation, and excellent biocompatibility [3].

The membranes can be derived from various natural sources, including platelets, cancer cells, and exosomes, each providing unique advantages [3–5]. Among these, platelets have emerged as especially relevant for addressing I/RI due to their capacity to target sites of vascular injury and escape immune surveillance [3], making them highly suitable for cell membrane-coated drug delivery approaches targeting I/RI. However, scalability, stability, and safety remain key challenges that require further investigation for the successful clinical application of these systems. Additionally, each drug delivery system has its own advantages and limitations, highlighting the need for tailored or combinatory strategies in I/RI therapy.

This review summarizes the application of platelet membrane-coated biomimetic drug delivery systems in the treatment of various I/RI, focusing on recent advancements, unresolved challenges, and a comparative analysis of each delivery system.

## Pathophysiological role of platelets in I/RI

Platelets, anucleate blood cells derived from megakaryocytes, are normally present in the blood at a count of 150–400 × 10^9 platelets/L [3, 6]. Their primary function is to maintain hemostasis by forming plugs at injury sites, releasing signaling molecules, and enhancing adhesion and clotting [7–8]. This process is mediated by its various membrane receptors, including integrins, seven-transmembrane receptors, immuno-globulin superfamily receptors, C-type lectins, and tetraspanins, all of which contribute to platelet activation and adhesion [3, 9–11]. For example, Integrin α2β1 and αIIbβ3 are key receptors in platelet adhesion and signaling through their interactions with collagen and fibrinogen, respectively [3]. C-type lectin P-selectin and immunoglobulin superfamily receptors, such as GPVI and FcR, also play key roles in promoting platelet aggregation and microthrombus formation at the site of injury [12]. Upon platelet activation, the expression of these surface receptors is significantly increased, which enhances platelet adhesion, aggregation, and overall activation.

**Fig. 1:**
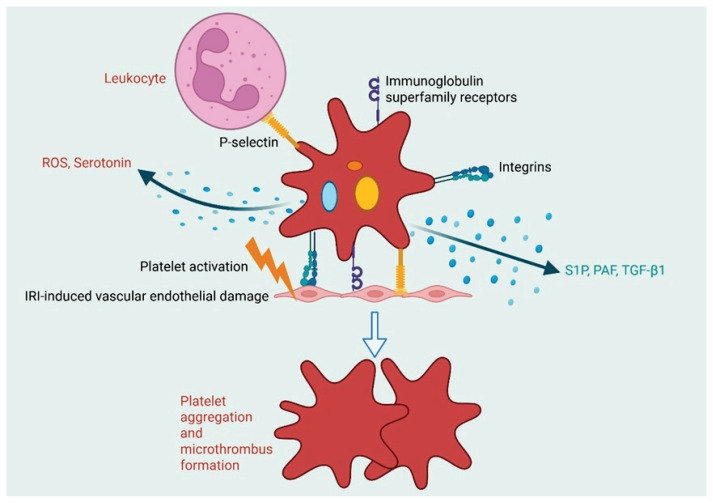
Pathophysiological roles of platelets in IRI. Endothelial damage from I/RI activates circulating platelets, leading to the upregulation of surface proteins such as integrins, immunoglobulin superfamily receptors, and C-type lectins (e.g., P-selectin). Activated platelets play dual roles: they exacerbate injury by promoting microthrombus formation (e.g., *via* platelet receptor GPVI), interacting with leukocytes and releasing pro-inflammatory mediators (e.g., ROS, serotonin); meanwhile, they also contribute to tissue repair by releasing protective factors such as S1P, low concentrations of PAF, and TGF-β1.

These properties of platelets make them essential to the pathogenesis of ischemia/reperfusion injury (I/RI), particularly in cardio-cerebrovascular conditions such as acute myocardial infarction (AMI), myocardial ischemia/reperfusion injury (MI/RI), and stroke [13]. As demonstrated in Fig. 1, in IR conditions, circulating platelets can be activated by I/RI-induced damage to the vascular endothelium [14]. This activation results in platelet adhesion to the reperfused endothelium, aggregation, and the formation of microthrombi [14]. In the post-I/RI tissue, activated platelets play a dual role in both tissue repair and aggravating the injury, as also illustrated in Fig. 1. On one hand, platelets can release protective molecules, including sphingosine-1-phosphate (S1P), low concentrations of platelet-activating factor (PAF), and transforming growth factor β1 (TGF-β1), during myocardial ischemia, which may contribute to cardioprotection [14]. On the other hand, activated platelets can aggravate I/RI by promoting microthrombus formation, interacting with leukocytes, and releasing pro-inflammatory molecules such as reactive oxygen species (ROS) and serotonin [15–16].

## Platelet membrane-camouflaged biomimetic drug delivery systems for I/RI

Given their pivotal role in I/RI pathophysiology, platelets have attracted significant interest as carriers for drug delivery systems aimed at mitigating I/RI-induced damage. The unique properties of platelet membranes confer several advantages for drug delivery, including: (1) excellent biocompatibility [16–17]; (2) natural targeting and adhesion properties, facilitated by the surface expression of specific proteins and receptors, such as P-selectin and collagen receptors (GPVI and FcR), which allow platelets to target damaged blood vessels [12, 16, 18–22]; (3) immune evasion mechanisms, where platelets use surface receptors like CD47 to camouflage themselves from immune system detection, enabling passive targeting of injured vascular endothelial cells [16, 23]. These properties position platelet membrane-camouflaged biomimetic drug delivery systems as more advantageous than other cell membrane-based drug delivery systems in the treatment of I/RI.

**Fig. 2:**
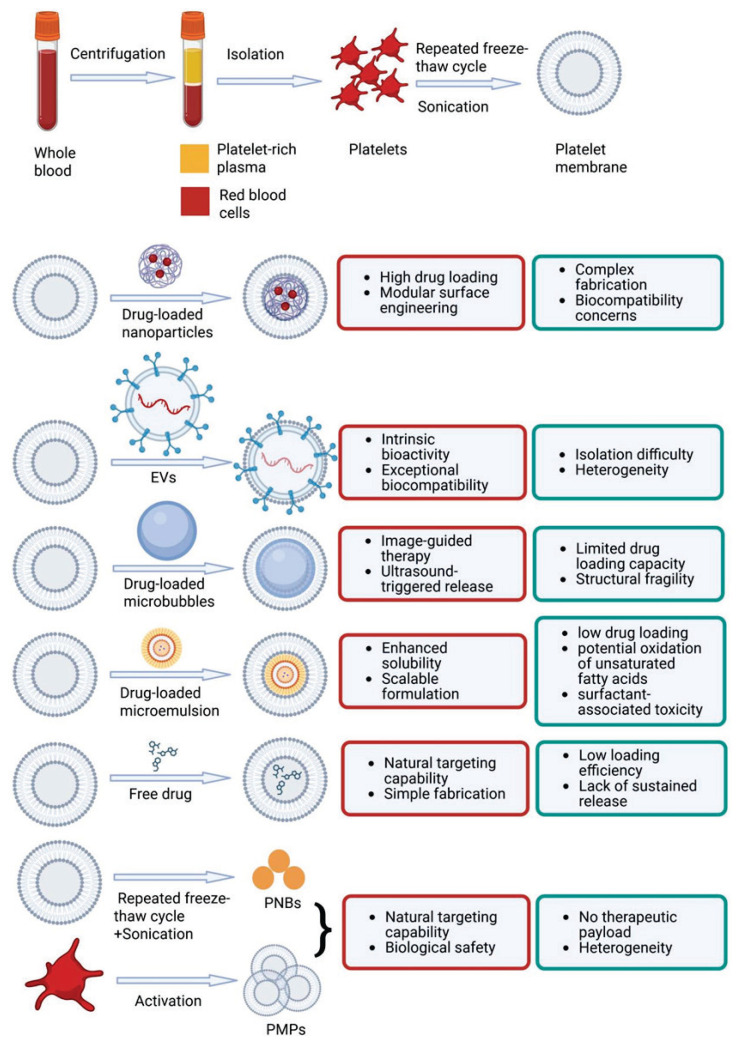
Preparation processes, advantages, and limitations of various types of platelet membrane biomimetic drug delivery systems

Using a simple and efficient top-down method, platelet membrane-camouflaged biomimetic drug delivery systems can be created through the sequential steps outlined in Fig. 2. This process involves isolating platelet membranes and integrating them into functional biomimetic delivery platforms by fusing them with various core carriers, such as drug-loaded nanoparticles, extracellular vesicles, microbubbles, and microemulsions, or by encapsulating free drugs (Fig. 2). A number of studies confirmed the potential of these systems in the treatment of diverse I/RI (Table 1).

## Studies utilizing platelet membrane-coated nanoparticles (PM-NPs)

PM-NPs combine immune evasion and tissue-targeting abilities of platelets with the drug-carrying capacity of nanoparticles for targeted therapy. The utilization of PM-NPs as potential therapeutic agents for I/RI has garnered significant attention in recent studies. These PM-NPs can be loaded with various therapeutic agents, such as antioxidants, anti-inflammatory agents, thrombolytics, neuroprotective compounds, and regenerative factors, to target ischemic and injured sites, enhancing treatment efficacy for conditions like MI/RI and stroke (Table 1). For example, a study demonstrated that coating mesoporous silica nanoparticles with platelet membranes enabled targeted delivery of SS31 peptide to ischemic cardiovascular sites, exerting antioxidant effects and alleviating MI/RI [24]. Another study explored the therapeutic potential of Poly(lactic-co-glycolic acid) (PLGA) nanoparticles camouflaged by platelet membrane vesicles (PMVs) to deliver microRNA inhibitors, which upregulate Nrf2, enhancing antioxidant defense, reducing oxidative stress, and mitigating inflammation, thereby providing protection against MI/RI [25]. Similarly, Cui *et al*. (2023) have explored the application of PM-NPs loaded with ginkgolide B, a compound with anti-inflammatory and antioxidative properties, for stroke treatment, demonstrating improved neural protection, reduced oxidative stress and inflammation, and enhanced motor recovery compared to conventional treatments [26]. Tan *et al*. (2021) also demonstrated that coating mesoporous silica nanospheres with platelet-like fusogenic liposomes, which are formed by hybridizing platelet membrane vesicles with cationic fusogenic liposomes, enabled targeted delivery of the anti-inflammatory miR-21 to inflammatory monocytes in mice with MI/RI, effectively mitigating inflammation and reducing tissue damage [27].

Furthermore, Yu *et al*. (2022) developed a biomimetic nanovesicle for ischemic stroke treatment, combining platelet membrane, near-infrared -mediated photothermal tissue plasminogen activator (tPA) release, and melanin nanoparticles for neuroprotection [28]. This approach accelerates thrombolysis in ischemic stroke, enhances tPA activity, and prevents reperfusion injury by scavenging free radicals and suppressing inflammation [28]. Similarly, Guo *et al*. (2022) developed a platelet-mimicking nanoparticle (PTPN) by combining a phenylboronic acid nanocarrier, antioxidant protocatechualdehyde (PC), and tPA, all encapsulated within a platelet membrane for thrombus targeting [29]. PTPN enabled precise delivery of tPA and PC, effectively reopened arteries, reduced oxidative stress, and protected heart mitochondria during MI/R [29]. Li *et al*. (2020) developed biomimetic nanocarriers by coating L-arginine-loaded magnetic nanoparticles with platelet membranes for targeted ischemic stroke treatment [30]. Guided by a magnetic field, these nanocarriers release L-arginine, enhancing nitric oxide production, vasodilation, and reducing platelet aggregation, improving blood flow and reperfusion [30]. Xu *et al*. (2019) developed a thrombin-responsive platelet biomimetic nanoplatform for dual-drug delivery in ischemic stroke treatment [31]. They loaded ZL006e, a neuroprotective agent, into acetal-modified dextran nanoparticles, which were then coated with platelet membranes and decorated with rtPA [31]. This system significantly improved stroke treatment by restoring cerebral blood flow and protecting neurons in the ischemic penumbra [31].

In addition, Wang *et al*. (2022) developed RGD-PLT@PLGA-FE, a biomimetic nanocarrier with platelet membranes decorated with Arg-Gly-Asp and a PLGA core encapsulating human fat extract, enabling targeted, sustained release at ischemic sites to promote angiogenesis, neurogenesis, and neurotrophic factor delivery for stroke recovery [32]. Su *et al*. (2019) also developed a platelet-inspired nanocell that integrates prostaglandin E2-modified platelet membranes with a PLGA nanoparticle-encapsulated core containing cardiac stromal cell-secreted factors, which significantly enhance targeted therapy for MI/RI by promoting tissue repair, improving cardiac function, and supporting regeneration [33].

These studies underscore the promising role of platelet membrane-coated nanoparticles in targeted drug delivery strategies for I/RI, offering effective therapeutic interventions in ischemic tissues.

## Studies utilizing platelet membrane-modified extracellular vesicles (P-EVs)

P-EVs inherited adhesive proteins and the natural targeting ability to injured vasculature from platelets while retaining the pro-angiogenic potential of EVs [34]. Several studies have explored the therapeutic potential of P-EVs for the treatment of I/RI, demonstrating promising outcomes in various preclinical models. One study constructed a biomimetic delivery system using P-EVs, which preferentially accumulated in the injured endothelium of ischemic hearts and enhanced the angiogenic potential of EVs in a MI/R mouse model [34]. In another study, P-EVs exhibited efficient targeting capabilities by enhancing uptake in human umbilical vein endothelial cells stressed by oxygen-glucose deprivation/reperfusion [35]. *In vivo*, they provided greater protection against MI/RI and reduced cardiac remodeling [35]. In addition, P-EVs mimicked platelet-monocyte binding in a MI/RI mouse model, allowing monocytes to carry them to the ischemic myocardium, where they released microRNAs to reduce inflammation and promote tissue repair [36]. These studies suggest the potential of P-EVs as promising therapeutic agents for the treatment of I/RI.

## Studies utilizing platelet membrane-coated biomimetic microbubbles

Microbubbles are gas-filled vesicles that can be used for imaging or drug delivery purposes. When the platelet membrane is used to coat microbubbles, they can selectively target areas of injury or inflammation by mimicking the biological behavior of platelets. Several studies have investigated the therapeutic potential of platelet membrane-coated biomimetic microbubbles for the treatment of I/RI, offering both diagnostic and therapeutic benefits. For example, two similar studies developed platelet membrane-coated biomimetic microbubbles (MB-pla) for noninvasive ultrasound imaging, demonstrating their potential for early MI/RI detection *in vitro* and *in vivo* [37–38]. Additionally, platelet membrane-mimicking hybrid microbubbles loaded with xenon (Xe-Pla-MBs) were designed for I/R-induced acute kidney injury (AKI) [39]. By delivering xenon to the injured site, Xe-Pla-MBs exhibited protective effects against I/R-induced AKI, potentially reducing renal senescence and preserving kidney function [39].

## Studies utilizing other platelet membrane-camouflaged biomimetic drug delivery systems

Beyond the methods mentioned above, other platelet membrane-coated biomimetic drug delivery systems have also been developed to treat I/RI. For example, Zou *et al*. (2022) developed platelet membrane-cloaked trimetazidine-loaded microemulsions (P/TMP-MEs) to enhance therapy for MI/RI [40]. Microemulsions are thermodynamically stable colloidal systems composed of oil, water, surfactants, and co-surfactants, which allow for improved drug solubility and absorption. By cloaking these microemulsions with platelet membranes, these P/TMP-MEs improved *in vivo* absorption and decreased non-targeted accumulation, thereby enhancing the efficacy of MI/R therapy [40].

PMVs derived from the plasma membranes of activated platelets may serve directly as versatile vehicles for drug delivery, as they retain the natural homing ability of platelets and can carry therapeutic agents themselves. In a study by Zhou *et al*. (2022), carvedilol, a beta-blocker with antioxidant properties, was encapsulated into PMVs. This formulation enhanced drug bioavailability and significantly improved cardiac function following MI/RI. [41].

## Studies utilizing PMVs and their derivatives

Direct application of PMVs and their derivatives may also help mitigate tissue damage and promote recovery in ischemic conditions. Platelet-derived microparticles (PMPs) are small vesicles (less than 1 μm) released from activated platelets, containing membrane components like receptors, lipids, and proteins. Ma *et al*. (2015) found that PMPs exhibited a protective effect against cardiac I/RI [42]. Additionally, Li *et al*. (2018) fabricated platelet-derived nanobubbles (PNBs) through repeated freeze-thawing and sonication of PMVs. These PNBs targeted brain occlusions from the onset of stroke, promoting microvascular recanalization and enabling real-time ultrasound-enhanced monitoring of ischemic lesion sites [43]. The mechanism behind these effects may be related to the platelet membrane components in both PMPs and PNBs, such as P-selectin and integrins, which facilitate interactions with target cells, activate cardioprotective signaling pathways, modulate inflammatory responses, and enhance tissue repair in ischemic conditions [44–45].

## Challenge and future perspectives

Cell membrane coating strategy is an emerging targeted delivery approach. Given the biological importance of platelets in I/RI, platelet membrane-camouflaged biomimetic drug delivery systems can be widely applied for both diagnostic and therapeutic purposes in I/RI [3]. However, research on platelet membrane-camouflaged biomimetic drug delivery systems is still in its early stages. One major challenge in scaling up coating is the substantial demand for platelet membranes, as 1 mg of PLGA nanoparticles requires more platelets than what is available in 10 ml of human blood [3, 46]. To address this shortage, infusing platelet membranes with phospholipids might be a promising solution [46]. Fusing platelet membranes with phospholipids, which can increase membrane quantity by several dozen to 500 times, has been shown to effectively target inflammatory monocytes and damaged endothelial cells in mice with MI/RI [27, 38], representing a promising solution to this shortage.

Another challenge is maintaining the structure and bioactivity of isolated platelet membranes due to their short shelf life [3, 46]. It has been shown that MB-pla exhibited the physicochemical properties of typical liposome-based microbubbles due to the hybridization of synthetic phospholipids and could stabilize *in vitro* for 3 hours, significantly outperforming pure cell membrane microbubbles [39]. Therefore, infusing platelet membrane with phospholipid might similarly enhance stability, which could help preserve both their structure and bioactivity.

Additionally, platelets contain pro-coagulant factors on their membrane. This raises safety concerns, as it remains unclear whether residual pro-coagulant factors on the platelet membrane may increase the risk of thrombosis. This potential risk requires further investigation before clinical translation. Furthermore, platelets can act as key regulators of inflammation [47–49], and it remains uncertain whether platelet membrane components could influence atherosclerosis progression by promoting inflammatory responses and contributing to plaque instability and thrombus formation.

For brain drug delivery, platelet membrane-camouflaged drug delivery systems are primarily used for ischemic stroke therapy due to their natural ability to target damaged blood vessels [47]. However, their effectiveness may be limited by challenges in crossing the blood-brain barrier (BBB), which has been partially addressed by modifying platelet membranes with TAT cell-penetrating peptides to enhance BBB penetration [31].

In addition, despite such advances targeting specific delivery barriers, it is important to recognize that these platelet membrane-based delivery strategies exhibit diverse strengths and limitations depending on their formulation and application (Fig. 2). PM-NPs combine synthetic cores with biological membranes, enabling high drug loading, immune evasion, and targeted delivery to injured vasculature [3]. Their modularity also allows for surface engineering and controlled release [3]. However, the fabrication process is relatively complex, and synthetic cores may introduce biocompatibility or regulatory concerns [3].

P-EVs offer exceptional biocompatibility and intrinsic bioactivity. They naturally carry therapeutic cargos such as microRNAs, which are responsible for modulating inflammation, promoting cell signaling, and facilitating tissue repair, all of which are critical in the context of I/RI [34–36]. Nonetheless, EVs are difficult to isolate in large quantities and often suffer from heterogeneity and limited scalability [50].

Platelet membrane-coated microbubbles provide an added advantage of image-guided therapy when used with ultrasound [37–39]. Their gaseous cores allow for real-time visualization and targeted disruption under insonation, making them suitable for localized thrombolysis or vascular repair [37–39]. However, their structural fragility, limited drug loading capacity and requirement for external ultrasound limit their application in systemic drug delivery [51].

Platelet membrane-cloaked microemulsion represents an emerging formulation where microemulsified drugs are encapsulated within a platelet membrane shell [40]. This system enhances drug solubility and platelet-mediated targeting while also offers easier formulation compared to EVs or solid-core nanoparticles [40]. However, their therapeutic application may be limited due to low drug loading, potential oxidation of unsaturated fatty acids which impacts efficacy and safety, and toxicity from surfactants [52].

Direct use of PMVs as drug carriers offers natural targeting capability and simple fabrication [41]. This approach avoids synthetic carriers and retains essential platelet surface markers [41]. However, this strategy may be constrained by low drug loading efficiency and the lack of sustained release. Moreover, current research on this method is limited, and its *in vivo* therapeutic performance, as well as its capacity to encapsulate diverse drug types rather than only specific ones, requires further investigation.

Direct use of PMVs without drug loading (e.g., PNBs or PMPs) leverages their biological safety and inherent targeting features [42–43]. While they show promise for immune modulation or tissue interaction, they lack drug payloads and exhibit heterogeneity, which may complicate their therapeutic application.

Therefore, while all platelet membrane-based systems share the common feature of platelet-derived targeting and immune evasion, their individual formulations present trade-offs in terms of fabrication complexity, scalability, drug payload, and functional versatility. A tailored and/or combined approach is necessary to optimally match the system with the specific demands of I/RI therapy. For instance, platelet membrane-coated microemulsions are well-suited for lipophilic drug delivery due to their high solubility and simple formulation [40], while PNBs are ideal for cerebral I/RI because of their small size and intrinsic bioactivity [43]. Furthermore, sequential use of two carriers may harness complementary strengths, for example, ultrasound-triggered platelet membrane-coated microbubbles to restore vessel patency, followed by p-EVs for tissue repair.

## Conclusion and Future Perspectives

Platelet membrane coating technology's greatest appeal lies in its ability to encapsulate drugs like a capsule, disguising them and mimicking natural biological processes to effectively target specific cells. Various platelet membrane-camouflaged biomimetic drug delivery systems, such as platelet membrane-modified nanoparticles, EVs, and microbubbles, have shown significant potential in I/RI treatment by facilitating the targeted delivery of therapeutic agents, such as antioxidants, anti-inflammatory drugs, thrombolytics, and neuroprotective compounds, to ischemic tissues. Despite these advancements, several challenges hinder the clinical translation of platelet membrane coating technology. Future research should focus on improving scalability for large-scale production, enhancing the stability and quality of platelet membrane-based formulations, ensuring their safety by studying potential side effects, and refining targeting strategies to increase therapeutic efficacy. Additionally, given the distinct strengths and limitations of each strategy, a tailored or combined approach will be essential to optimally align each system with the specific demands of I/RI therapy.

## Figures and Tables

**Table 1:** Summary of platelet membrane-camouflaged biomimetic drug delivery systems for I/RI.

Sr. No	Drug delivery strategies	Cargo	IRI type	Key advances	References
**1**	Platelet membrane-coated mesoporous silica nanocarrier	SS31 peptide-	MI/RI	Targeted drug delivery, improvement of MI/RI therapy.	[24]
**2**	Platelet membrane vesicle-camouflaged PLAG nanoparticles	MicroRNA inhibitors	MI/RI	Targeted drug delivery, improvement of MI/RI therapy.	[25]
**3**	Platelet-derived nanoparticles	Ginkgolide B	Ischemic stroke	Targeted drug delivery, improvement of ischemic stroke therapy.	[26]
**4**	Platelet membrane-coated mesoporous silica nanospheres	miR-21	MI/RI	Targeted delivery of miR-21, improvement of MI/RI therapy.	[27]
**5**	Platelet membrane-encapsulated melanin nanovesicle	tPA	Ischemic stroke	Targeted drug delivery, improvement of ischemic stroke therapy.	[28]
**6**	Platelet membrane-coated phenylboronic acid nanoparticle	Protocatechualdehyde and tPA	MI/RI	Targeted drug delivery, improvement of MI/RI therapy.	[29]
**7**	Platelet membrane magnetic nanocarriers	l-arginine	Ischemic stroke	Targeted drug delivery, improvement of ischemic stroke therapy.	[30]
**8**	rtPA decorated Platelet membrane-coated acetal-modified dextran polymer nanoparticles	ZL006e	Ischemic stroke	Targeted drug delivery, improvement of ischemic stroke therapy.	[31]
**9**	RGD-decorated platelet membrane-coated PLGA nanocarrier	Human fat extract	Ischemic stroke	Targeted drug delivery, improvement of ischemic stroke therapy.	[32]
**10**	Prostaglandin E2 modified platelet membrane-coated PLGA nanoparticles	Cardiac stromal cell-secreted factors	MI/RI	Targeted drug delivery, improvement of MI/RI therapy.	[33]
**11**	Platelet membrane modified EVs	-	MI/RI	Targeted delivery of EVs, improvement of MI/RI therapy.	[34]
**12**	Platelet membrane-fused circulating EVs	-	MI/RI	Targeted delivery of EVs, improvement of MI/RI therapy.	[35]
**13**	Platelet membrane modified EVs	microRNAs	MI/RI	Targeted delivery of EVs, improvement of MI/RI therapy.	[36]
**14**	Platelet membrane-coated biomimetic microbubbles	-	MI/RI	Early detection of MI/RI	[37]
**15**	Platelet membrane-coated PLGA microbubbles	-	MI/RI	Early detection of MI/RI	[38]
**16**	Platelet membrane-mimicking hybrid microbubbles	Xenon	IR-induced AKI	Targeted drug delivery, Improvement of IR-induced AKI therapy.	[39]
**17**	Platelet membrane-cloaking microemulsion	Tetramethylpyrazine	MI/RI	Targeted drug delivery, improvement of MI/RI therapy.	[40]
**18**	Platelet membrane vesicles	Carvedilol	MI/RI	Targeted drug delivery, improvement of MI/RI therapy.	[41]
**19**	Platelet-derived microparticles	-	MI/RI	Protects against MI/RI.	[42]
**20**	Platelet-derived nanobubbles	-	Ischemic stroke	Targeted drug delivery, improvement of ischemic stroke therapy, ultrasound-enhanced imaging for real-time monitoring.	[43]
